# From bench to bedside: investigating SGLT2 inhibitors as a novel strategy against chemotherapy-induced cardiomyopathy

**DOI:** 10.3389/fcvm.2025.1647747

**Published:** 2025-09-22

**Authors:** Rade Jibawi Rivera, Faris Muaref, Sulaiman Paika, Abdullah Ruyyashi, Christopher S Gondi

**Affiliations:** ^1^ Internal Medicine, University of Illinois Chicago College of Medicine, Chicago, IL, United States; ^2^ Internal Medicine, Mayo Clinic Minnesota, Rochester, MN, United States; ^3^ Internal Medicine, Nova Southeastern University Dr Kiran C Patel College of Allopathic Medicine, Fort Lauderdale, FL, United States; ^4^ Internal Medicine, Abrazo Health Care, Phoenix, AZ, United States

**Keywords:** cardiotoxicity, anthracyclines, SGLT2 inhibitors, heart failure prevention, cardioprotection, ACE-inhibitors, β-blockers cardiotoxicity, β-blockers

## Abstract

**Background:**

Anthracyclines are essential components of chemotherapeutic regimens for a broad spectrum of malignancies, yet their utility is constrained by cumulative, dose-dependent cardiotoxicity, often culminating in non-ischemic cardiomyopathy and heart failure. The pathogenesis involves oxidative stress, mitochondrial dysfunction, and topoisomerase IIβ–mediated DNA damage in cardiomyocytes. While ACE inhibitors and angiotensin receptor blockers (ARBs) have demonstrated modest cardioprotective effects, the efficacy of newer heart failure therapies remains underexplored. Sodium-glucose co-transporter-2 (SGLT2) inhibitors, endorsed as Class I therapy for heart failure with reduced ejection fraction (HFrEF) per 2022 AHA/ACC/HFSA guidelines, have shown robust cardioprotective effects in large cardiovascular outcomes trials. However, their potential to prevent or attenuate anthracycline-induced cardiotoxicity has not been systematically evaluated. This study aimed to assess preclinical and clinical evidence supporting their use in anthracycline-exposed populations.

**Methods:**

A systematic review was conducted by PRISMA 2020 guidelines. Comprehensive searches of major medical databases and clinical trial registries were performed through March 2025. Eligible studies investigated SGLT2 inhibitors, β-blockers, or ACE inhibitors in adult patients receiving anthracycline-based chemotherapy or in animal models replicating this exposure. Primary outcomes included changes in LVEF, GLS, and incidence of heart failure. Studies involving pre-existing heart failure or non-anthracycline-related cardiotoxicity were excluded.

**Results:**

Preclinical studies (*n* = 4) consistently demonstrated that SGLT2 inhibitors mitigated cardiomyocyte injury, fibrosis, and oxidative stress, preserving cardiac function in anthracycline-exposed models. In one study, LVEF was significantly higher in animals treated with SGLT2 inhibitors (61.3% ± 11%) vs. controls (49.2% ± 8%, *p* = 0.007). Additional studies corroborated reduced histopathological damage and improved myocardial performance. No clinical trials to date have specifically assessed SGLT2 inhibitors in oncology populations. Nevertheless, major cardiovascular trials (e.g., EMPA-REG OUTCOME, DECLARE-TIMI 58) have demonstrated substantial reductions in heart failure events among non-cancer cohorts. In contrast, ACE inhibitors and β-blockers have shown variable efficacy during chemotherapy, with inconsistent findings across studies.

**Conclusions:**

SGLT2 inhibitors exhibit consistent cardioprotective effects in preclinical models of anthracycline cardiotoxicity and possess well-established efficacy in broader cardiovascular populations. These findings underscore the critical need for prospective trials evaluating their safety and therapeutic potential in cardio-oncology, with implications for reshaping current preventive strategies.

**Systematic Review Registration:**

PROSPERO [1056661].

## Introduction

Anthracyclines are a class of chemotherapeutic agents widely used to treat various cancers, including hematologic malignancies and solid tumors. These drugs have been shown to be highly effective due to their ability to interfere with DNA replication and transcription, making them particularly useful against rapidly proliferating cancer cells. However, despite their efficacy, anthracyclines have been associated with dose-dependent cardiotoxicity, which can lead to long-term complications such as congestive heart failure (1). The clinical challenge lies in balancing their potent anticancer effects with the risk of adverse cardiovascular outcomes.

The cardiotoxicity of anthracyclines is believed to arise from multiple mechanisms, including the inhibition of topoisomerase II enzymes, oxidative stress, and mitochondrial dysfunction. While these drugs target topoisomerase II*α* (TOP2A) in tumor cells, they also affect topoisomerase IIβ (TOP2B) in cardiac tissue, leading to DNA damage and impaired cellular function (2). Additionally, anthracyclines have been shown to promote the formation of reactive oxygen species, subsequently contributing to oxidative damage and cardiomyocyte apoptosis (3). These effects may result in both acute and chronic cardiac complications.

Despite their limitations, anthracyclines remain a cornerstone of cancer management due to their broad-spectrum activity against various cancers. However, the challenge moving forward is in refining treatment strategies that maximize therapeutic benefit while minimizing long-term toxicity. Given the persistent challenge of anthracycline-induced cardiotoxicity, considerable research has focused on identifying effective prophylactic strategies to safeguard cardiovascular health in cancer patients. Dexrazoxane is an FDA-approved agent used for cardioprotection in high-risk patients receiving anthracyclines, but recent attention has shifted toward heart failure medications such as ACE inhibitors, β-blockers, and SGLT2 inhibitors as promising alternatives or adjuncts (4). Historically, certain pharmacological agents have shown promise in this regard. Among these pharmacological agents are angiotensin-converting enzyme (ACE) inhibitors and β-blockers.

ACE inhibitors and β-blockers together form the backbone of chronic heart failure treatment, as both classes of drugs have been shown to reduce morbidity and mortality (5). β-blockers work by blocking β-adrenergic receptors, reducing the effects of adrenaline associated hormones on the heart. ACE inhibitors help manage heart failure and hypertension by blocking the conversion of angiotensin I to angiotensin II, a vasoconstrictor. In patients undergoing chemotherapy, ACE inhibitors and β-blockers have been studied for their ability to reduce left ventricular ejection fraction (LVEF) decline, a critical clinical determinant in the diagnosis of heart failure. Randomized controlled trials such as the Prevention of Cardiac Dysfunction During Adjuvant Breast Cancer Therapy (PRADA) (6) and the preventiOn of left Ventricular dysfunction with Enalapril and caRvedilol in patients submitted to intensive ChemOtherapy for the treatment of Malignant hEmopathies (OVERCOME) (7) have demonstrated that these medications may significantly reduce the incidence of heart failure events and LVEF decline compared to placebo.

**Figure 1:**
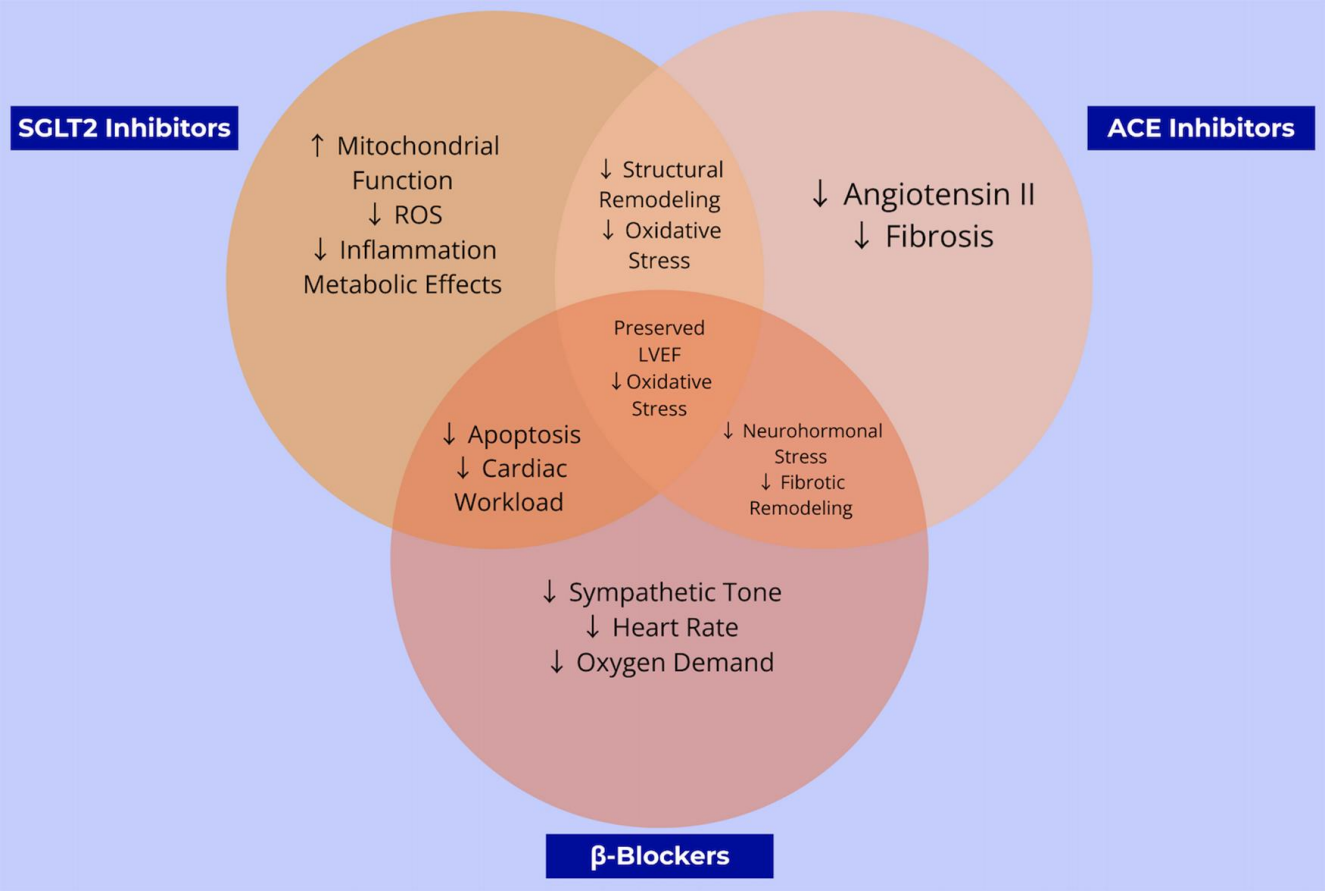
This schematic shows how β-blockers reduce sympathetic activation, heart rate, and myocardial oxygen demand to limit ischemic injury and oxidative stress. Together, these pathways help preserve LVEF and reduce the incidence of heart failure in patients treated with anthracyclines.

**Figure 2:**
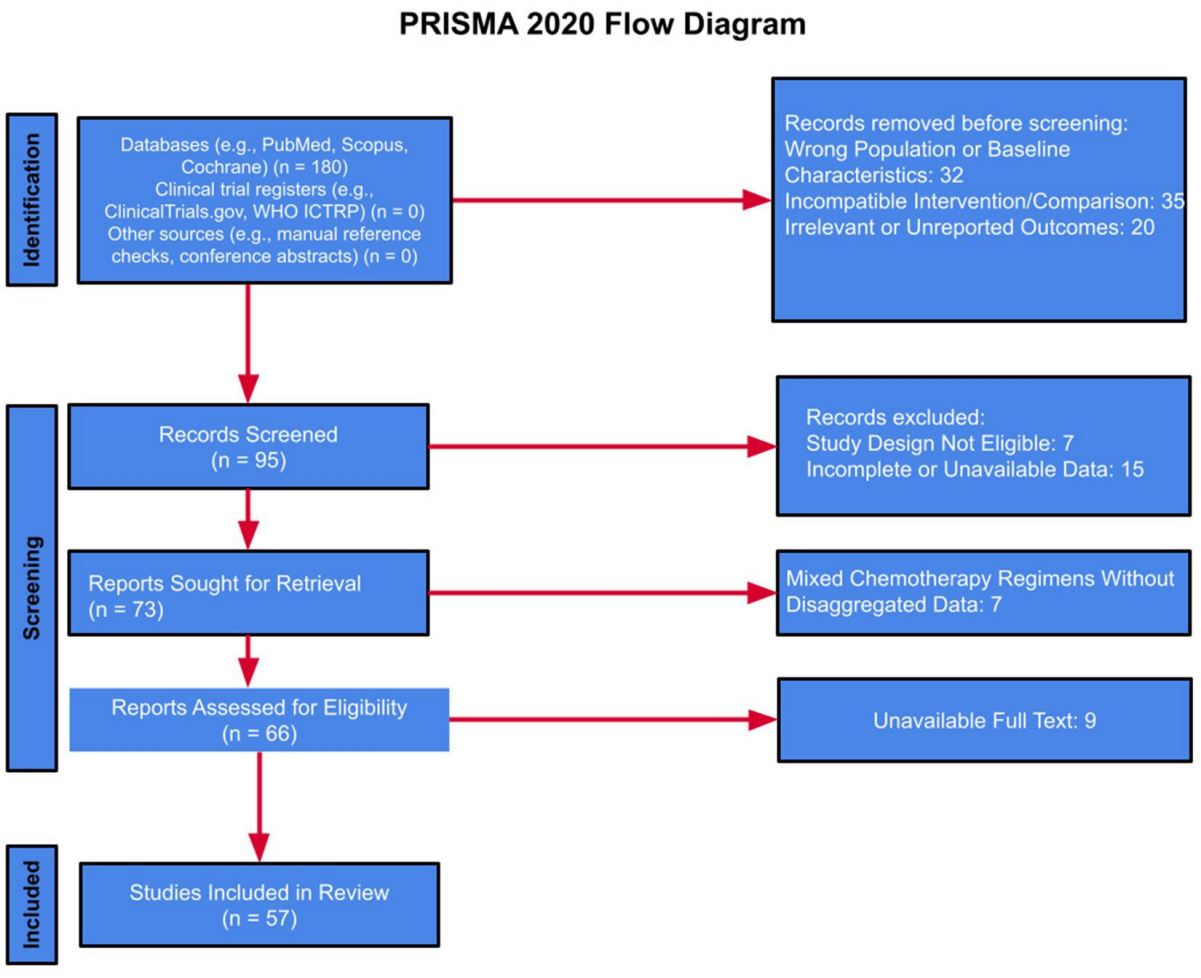
This figure illustrates the mechanisms by which ACE inhibitors lower angiotensin II and aldosterone levels, leading to vasodilation, decreased afterload, and reduced wall stress. These effects help prevent remodeling and fibrosis, ultimately preserving LVEF and minimizing heart failure risk.

ACE inhibitors and β-blockers constitute an essential component of guideline-directed medical therapy (GDMT), the first-line therapeutic strategy for heart failure management. This regimen has demonstrated robust efficacy in attenuating chemotherapy-induced cardiotoxicity by preserving left ventricular systolic function (8). ***To clarify these mechanisms,*** Figure 1 ***illustrates the key pathways by which β-blockers exert cardioprotective effects, and*** Figure 2 ***details the parallel mechanisms for ACE inhibitors, specifically in the context of anthracycline-induced cardiotoxicity*.**

Despite their designation as the therapeutic gold standard, concerns remain regarding their utility in the primary prevention of heart failure, as their role has been predominantly centered on the management of established cardiac disease. It is in this context that Sodium-glucose cotransporter 2 (SGLT2) inhibitors have emerged and shown comparable cardioprotective effects, offering a promising alternative or adjunct strategy in the context of chemotherapy-induced cardiotoxicity.

**Figure 3:**
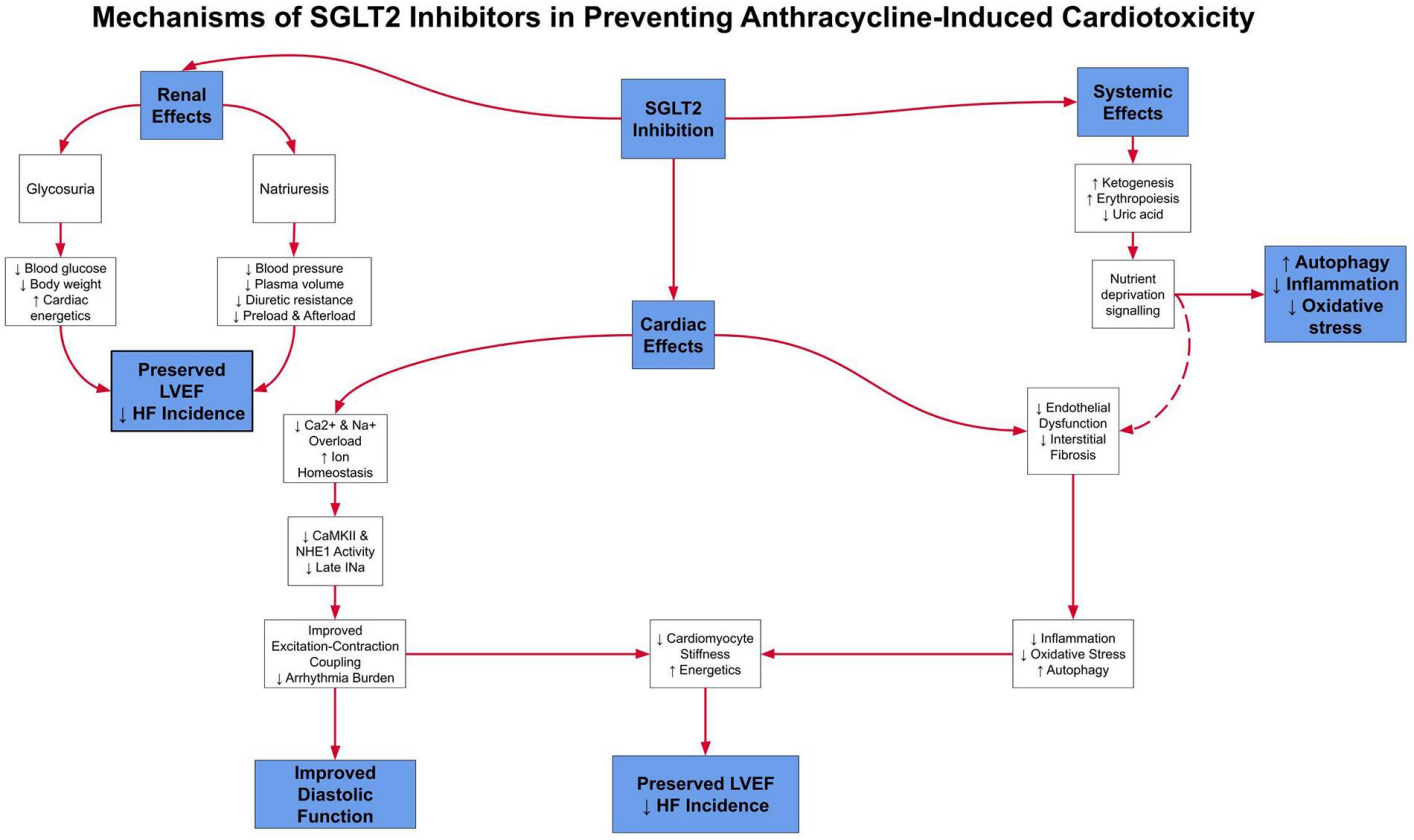
This schematic outlines the multiple pathways through which SGLT2 inhibitors exert cardioprotective effects, including natriuresis, improved myocardial energetics, and decreased oxidative stress. These complementary effects may provide added protection against anthracycline-induced cardiomyopathy.

SGLT2 inhibitors are primarily utilized in the management of type 2 diabetes mellitus. Their mechanism of action involves inhibition of SGLT2 in the proximal renal tubules, thereby decreasing renal glucose reabsorption and enhancing urinary glucose excretion, ultimately leading to improved glycemic control. Beyond their glucose-lowering effects, SGLT2 inhibitors have recently shown significant cardiovascular and renal benefits. They have been shown to reduce heart failure hospitalizations, cardiovascular mortality, and the progression of renal disease. Clinical trials, including EMPA-REG OUTCOME trial (EMPA-REG OUTCOME), Canagliflozin Cardiovascular Assessment Study (CANVAS), and Dapagliflozin Effect on Cardiovascular Events-Thrombolysis in Myocardial Infarction 58 (DECLARE-TIMI 58) (9, 56), have demonstrated that these agents provide cardiovascular protection in both diabetic and non-diabetic populations, with particular benefit in patients with heart failure. The cardioprotective mechanisms of SGLT2 inhibitors are diverse, including blood pressure reduction, natriuresis, and decreased oxidative stress. In models of anthracycline-induced cardiomyopathy, they have also been shown to mitigate inflammation and improve myocardial metabolism (10). Figure 3 summarizes these mechanisms, highlighting how SGLT2 inhibition may address gaps left by ACE inhibitors and β-blockers.

**Figure 4:**
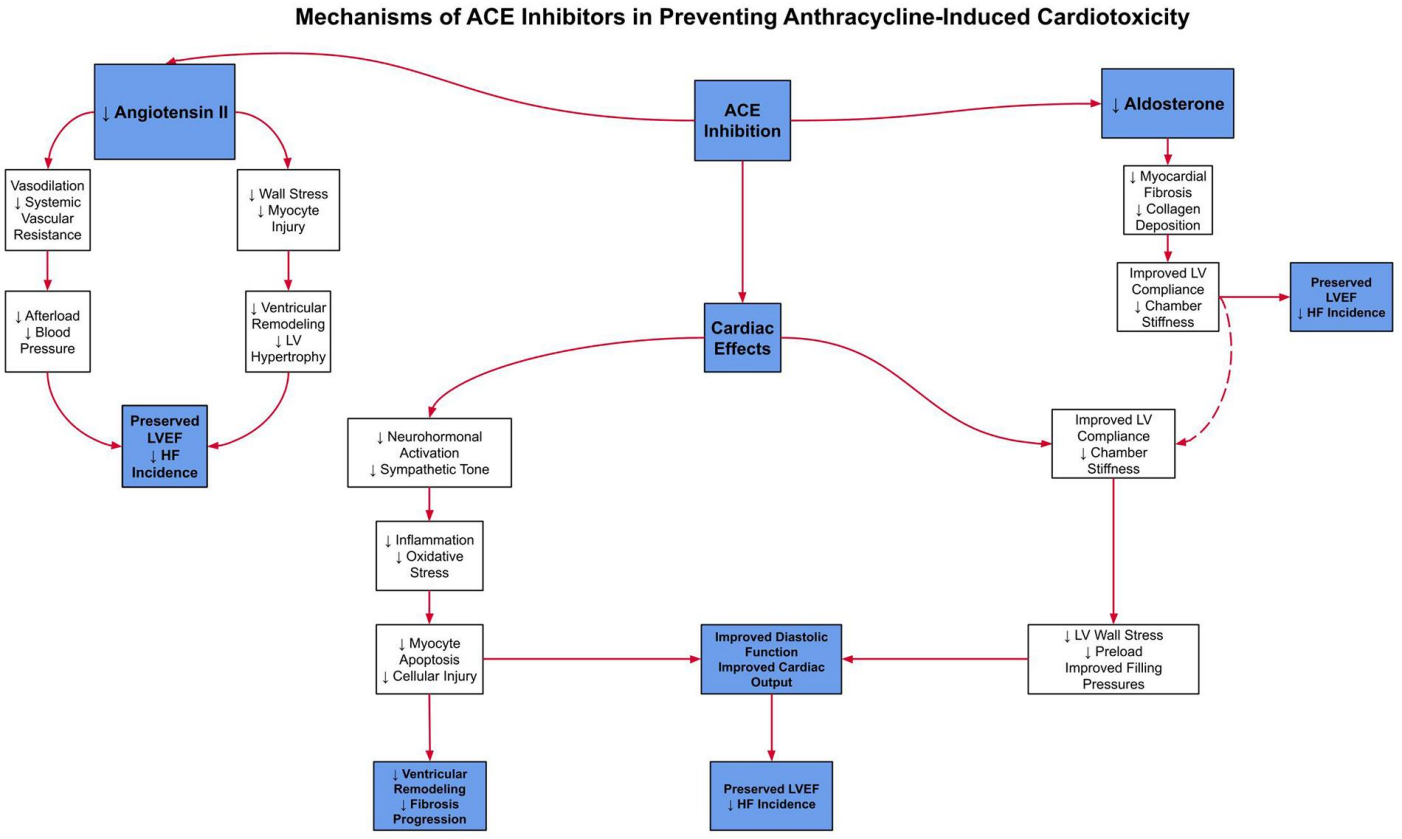
This venn diagram compares and contrasts the overlapping and unique cardioprotective mechanisms of β-blockers, ACE inhibitors, and SGLT2 inhibitors. The shared and distinct pathways highlight opportunities for combined or alternative strategies to prevent chemotherapy-induced heart failure.

Taken together, these complementary and overlapping pathways are illustrated in Figure 4, which compares and contrasts the molecular actions of SGLT2 inhibitors, ACE inhibitors, and β-blockers in the context of anthracycline-induced cardiotoxicity.

These complementary and overlapping mechanisms are illustrated in Figure 1, which compares the molecular actions of SGLT2 inhibitors, ACE inhibitors, and β-blockers in the context of anthracycline-induced cardiotoxicity. While all three classes share the common goal of preserving cardiac function, SGLT2 inhibitors uniquely address mitochondrial energetics and systemic inflammation, whereas ACE inhibitors and β-blockers act primarily via hemodynamic and neurohormonal pathways. This divergence in mechanistic targets may explain the broader cardioprotective effects observed with SGLT2 inhibitors in both preclinical and clinical models, particularly in patients without established heart failure.

Despite promising evidence, there remains a critical lack of head-to-head trials directly comparing SGLT2 inhibitors with standard cardioprotective agents for patients receiving anthracycline chemotherapy. Current cardio-oncology guidelines offer limited guidance for patients without overt heart failure or diabetes, leaving clinicians with few evidence-based options to protect cardiovascular health in this unique population. By addressing these gaps, this synthesis highlights the timely need for rigorous evaluation of SGLT2 inhibitors as a novel preventive strategy in non-diabetic, anthracycline-exposed cancer patients.

The objective of this study is to determine whether SGLT2 inhibitors can provide superior or complementary cardiovascular protection when compared to ACE inhibitors and β-blockers in preventing cardiomyopathy in patients starting anthracycline chemotherapy by evaluating the effects of SGLT2 inhibitors on the incidence of HF and changes in LVEF in this high-risk population.

To date, no head-to-head clinical trials have compared SGLT2 inhibitors with standard cardioprotective agents in patients undergoing anthracycline-based chemotherapy. Moreover, existing guidelines provide limited direction for cardio-oncology patients without overt heart failure or diabetes. This systematic review addresses a critical evidence gap by synthesizing the current preclinical and clinical landscape and evaluating the potential role of SGLT2 inhibitors in non-diabetic, anthracycline-exposed oncology populations. As survival in cancer patients improves, timely identification of novel preventive strategies is essential to mitigate long-term cardiovascular morbidity.

## Methods

This study was designed as a systematic review of preclinical and clinical studies investigating the cardioprotective effects of SGLT2 inhibitors, ACE inhibitors, and β-blockers in patients receiving anthracycline-based chemotherapy. The primary objective was to compare the efficacy of these pharmacologic agents in preventing heart failure and preserving LVEF in patients without pre-existing heart failure (baseline LVEF >50%).

### Search strategy

A comprehensive literature search was conducted using databases including PubMed, Embase, Scopus, and the Cochrane Library. To ensure a more comprehensive review, additional searches were conducted using clinical trial registries, including ClinicalTrials.gov, the World Health Organization International Clinical Trials Registry Platform (WHO ICTRP), and the EU Clinical Trials Register, as well as sources of unpublished research such as conference proceedings from the American Heart Association and the American Society of Clinical Oncology, dissertations, and institutional repositories. Keywords included combinations of terms such as “anthracycline”, “doxorubicin”, “cardiotoxicity”, “heart failure”, “SGLT2 inhibitors”, “empagliflozin”, “dapagliflozin”, “β-blockers”, “ACE inhibitors,” “LVEF”, and “cardioprotection”. The search was limited to articles published in English up to March 2025. These searches were conducted on March 3rd–6th and 10th–12th, 2025.

### Study selection criteria

**Figure 5:**
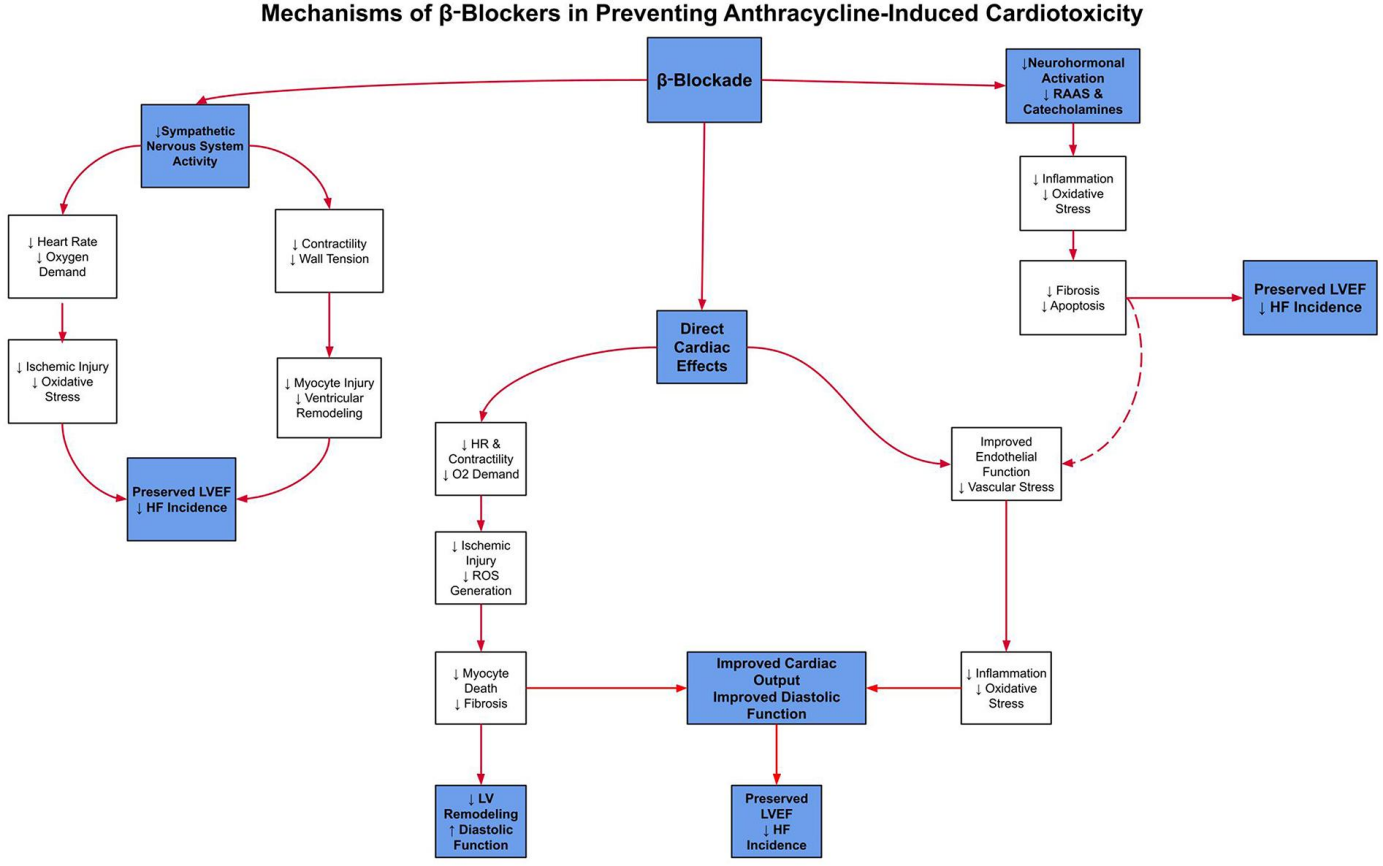
PRISMA study selection summary: summary of the study selection process, including the number of records identified, screened, excluded, and included in the final review, based on PRISMA guidelines.

Data were extracted independently by two authors, followed by full-text review to confirm eligibility (Figure 5). Discrepancies in study inclusion were initially addressed through structured discussion between the reviewers. If consensus could not be reached, a third senior reviewer was consulted to adjudicate the final decision, in accordance with PRISMA 2020 guidelines.

Eligible studies included randomized controlled trials, cohort studies, observational studies, and preclinical investigations evaluating cardiac outcomes in the context of anthracycline therapy. Meta-analyses and systematic reviews were also included to synthesize broader evidence. Studies were included if they reported outcomes related to heart failure incidence, changes in LVEF, global longitudinal strain (GLS), or cardiac biomarkers in response to SGLT2 inhibitors, ACE inhibitors, or β-blockers. Trials involving patients with pre-existing heart failure or reduced baseline LVEF (<50%) were excluded unless deemed relevant for comparative context, such as those examining populations with similar diagnoses to the target group or, alternatively, populations without any diagnoses, allowing for meaningful comparison. Additionally, studies not originally published in English, editorials, and those lacking quantitative results were excluded. Data extracted from eligible studies included author, year, study design, sample size, population characteristics, type and dosage of anthracycline administered, cardioprotective intervention used, follow-up duration, and specific cardiac outcomes, including changes in LVEF, incidence of symptomatic heart failure (as defined by the original study), GLS, and cardiac biomarkers such as NT-proBNP and troponin levels.

### Bias assessment

Risk of bias was assessed using the Risk Of Bias In Non-randomized Studies of Interventions (ROBINS-I) tool, which is specifically designed to evaluate bias in studies comparing two or more health interventions. This tool examines seven domains: confounding, participant selection, intervention classification, deviations from intended interventions, missing data, outcome measurement, and selection of reported results (11). Each study was independently evaluated by two authors; any disagreements in domain ratings or overall bias assessments were resolved through discussion. If consensus could not be reached, a third reviewer with expertise in systematic review methodology adjudicated the final decision.

Each domain was rated as having low, moderate, or serious risk of bias, or categorized as having insufficient information. Most randomized controlled trials were judged to have a low risk of bias using the Cochrane ROB 2.0 tool, except for seven trials—Bosch et al. (7), Guglin et al. (12), Gulati et al. (6), Livi et al. (13), SOLVD Investigators (1991), SOLVD Investigators (14), and Rode et al. (15)—which were rated as having some concerns due to unclear allocation concealment and/or incomplete reporting of chemotherapy type and/or dosage.

### Review conduct and standards

This systematic review was conducted in accordance with the PRISMA 2020 guidelines (16), which provide a 27-item checklist and flow diagram to enhance the transparency and completeness of reporting in systematic reviews. The review protocol was prospectively registered with PROSPERO (1056661) to ensure methodological transparency and to minimize the risk of bias.

### Data synthesis and statistical interpretation

Due to significant heterogeneity across included studies, ranging from differences in study design, patient populations, intervention dosages, and outcome measurement methods, a formal meta-analysis was not feasible. Therefore, data analysis was approached through qualitative synthesis, focusing on a narrative summary supported by structured quantitative interpretation.

Studies were grouped into two primary categories based on pharmacological class: SGLT2 inhibitors formed one group, and ACE inhibitors and β-blockers were combined into a second group. These groups were then further stratified by study design (e.g., randomized controlled trials, cohort studies, preclinical investigations). Quantitative results for primary and secondary outcomes were systematically extracted, including LVEF changes (e.g., mean difference, percent change), heart failure incidence [e.g., Hazard Rations (HRs), Relative Risks (RRs), absolute event rates], mortality rates, and, where available, CIs and p-values.

Statistical interpretation focused on several dimensions. First, we assessed the consistency and direction of effect, whether outcomes such as LVEF preservation or reductions in heart failure incidence showed uniform benefit across studies within each pharmacologic group. We also evaluated the magnitude of effect, considering reported HRs, RRs, and mean differences to determine clinical relevance. Precision and statistical significance were assessed by examining confidence intervals and p-values, with attention to studies reporting non-significant results or wide intervals that might affect the robustness of findings. To understand variability, we contextualized outcomes based on follow-up duration, anthracycline dosage, and intervention intensity, which allowed us to assess the generalizability of observed effects across clinical scenarios. Lastly, comparative trends were examined between the SGLT2 inhibitor and ACE inhibitor/β-blocker groups, highlighting where one class demonstrated greater or more statistically consistent cardioprotective benefit.

No *de novo* statistical analyses were performed. Key findings and extracted data are presented in descriptive tables and figures to illustrate observed patterns and comparative insights.

## Results

A total of 52 studies were included in this analysis, comprising randomized controlled trials, cohort studies, and preclinical investigations. Additionally, six meta-analyses were reviewed, encompassing 52 trials. Sample sizes varied widely, ranging from 9 to 10,142 participants, with follow-up durations spanning 3 days to 10 years. The studies primarily focused on patients receiving anthracycline-based chemotherapy (e.g., doxorubicin, epirubicin, idarubicin) for cancers such as breast cancer, lymphoma, and leukemia. Several studies also examined the cardioprotective effects of various drugs in patients without cancer. Studies prioritizing populations without pre-existing heart failure were included; however, this inclusion criterion was applied with greater flexibility in the evaluation of ACE inhibitors and β-blockers.

SGLT2 inhibitors, including empagliflozin and dapagliflozin, were assessed as potential cardioprotective agents, with comparators such as placebo, standard heart failure therapies (e.g., carvedilol, ACE inhibitors), or no intervention. Primary outcomes varied but frequently included heart failure incidence, LVEF changes, and GLS assessments. The diversity in study designs, patient populations, and intervention strategies highlights the growing interest in SGLT2 inhibitors as cardioprotective agents in anthracycline-treated patients.

Among the 55 total studies reviewed, 25 studies specifically examined the effects of SGLT2 inhibitors on heart failure outcomes In Table 1, a major trial showed canagliflozin significantly reduced the risk of major cardiovascular events and kidney failure, showing a hazard ratio of 0.68 (95% CI, 0.49–0.94) for primary prevention and 0.85 (95% CI, 0.69–1.06) for secondary prevention (17). Similarly, SGLT2 inhibitors, compared to other glucose-lowering drugs, were associated with a lower incidence of heart failure (HR, 0.61; 95% CI, 0.51–0.73; *P* < 0.001) (18). In preclinical models, empagliflozin preserved left ventricular function despite anthracycline exposure. Control mice exhibited a significant decline in ejection fraction, while empagliflozin-treated mice maintained stable cardiac function (*P* = 0.011) (19). Similarly, in the EMPA-DOXO (empagliflozin-doxorubicin) group, heart failure incidence was significantly reduced (*P* < 0.001), with ejection fraction improvement from 81.2 ± 2.5% to 88.3 ± 2.3% (*P* < 0.05) (20). Other studies confirmed LVEF recovery and fractional shortening (FS) in DOX + EMPA groups (*P* = 0.06) (21) and improved LVEF (61.3 ± 11% vs. 49.24 ± 8%, *P* = 0.007) in treated groups (22). Furthermore, Ejection fraction decline was significantly worse in DOX-only groups compared to SGLT2 inhibitor-treated groups (66.00% ± 9.98% vs. 78.80% ± 2.57%, 80.90% ± 3.10%, and 74.00% ± 4.03%) (23).

**Table 1:** Summary of clinical and meta-analysis studies involving SGLT2 inhibitors.

Study ID	Design	Intervention & duration	Outcomes
Anker et al. 2021 (39)	RCT	Empagliflozin 10 mg QD; 26.2 months	↓ HF hospitalization (HR 0.73; 95% CI, 0.61–0.88; *p* < 0.001)
Das et al. 2024 (40)	Meta-analysis	Dapagliflozin 10 mg QD; 7.5 months	↑ NYHA functional class (log OR 1.3; 95% CI, 0.37–2.23; *p* = 0.01)
Byrne et al. 2017 (19)	RCT	Empagliflozin 10 mg/kg; 0.5 months	EF preserved vs decline in controls
Fitchett et al. 2016 (8)	RCT	Empagliflozin 10/25 mg QD; 36 months	↓ HF hospitalization or CV death (HR 0.66; CI 0.55–0.79; *p* < 0.001)
Kosiborod et al. 2017 (18)	Meta-analysis	Various SGLT2i; various durations	↓ HF incidence vs other drugs (HR 0.61; CI 0.51–0.73; *p* < 0.001)
Mahaffey et al. 2018 (41)	RCT	Canagliflozin 100 mg; 60 months	↓ primary endpoint vs placebo (HR 0.86; CI 0.75–0.97; *p* < 0.001)
Mahaffey et al. 2019 (17)	RCT	Canagliflozin 100 mg QD; 31.4 months	↓ primary (HR 0.68) and secondary (HR 0.85) endpoints
McMurray et al. 2019 (42)	RCT	Dapagliflozin 10 mg QD; 18.2 months	↓ HF events (10% vs 13.7%; HR 0.70; CI 0.59–0.83)
Shi et al. 2017 (43)	RCT	Canagliflozin; 0.1 months	↓ BNP by 35%; stepwise BNP reduction (*p* < 0.05)
Zannad et al. 2020 (44)	Meta-analysis	Empagliflozin 10 mg QD; 16 months	↓ HF hospitalization or CV death (HR 0.75; CI 0.68–0.84; *p* < 0.0001)
Zelniker et al. 2019 (45)	Meta-analysis	Various SGLT2i; 3 months	↓ HF hospitalization/CV death (HR 0.77; CI 0.71–0.84; *p* < 0.0001)
Zinman et al. 2015 (46)	RCT	Empagliflozin 10/25 mg QD; 37.2 months	↓ HF hospitalization by 35%

In a large, propensity-matched observational study of 1,412 anthracycline-treated patients without pre-existing heart failure, Fath et al. found that SGLT2 inhibitors were associated with significantly reduced rates of new-onset heart failure (HR: 0.15, 95% CI: 0.07–0.29) and arrhythmias (HR: 0.40, 95% CI: 0.23–0.69), without increased risk of renal dysfunction or mortality, further supporting their potential for cardioprotection in this high-risk population (24). Similarly, Bhatti et al. demonstrated that baseline SGLT2 inhibitor use in a large cohort of over 17,000 adults with cancer and type 2 diabetes was associated with a significantly reduced risk of cancer therapy–related cardiac dysfunction (HR: 0.76; 95% CI: 0.69–0.84), as well as lower rates of heart failure exacerbations and all-cause mortality (25). The cardioprotective effects of SGLT2 inhibitors across both animal models and clinical datasets are summarized in Table 2, highlighting their potential role in heart failure prevention and LVEF preservation.These findings demonstrate consistent improvements in both functional cardiac measures and clinical endpoints, supporting SGLT2 inhibitors as a mechanistically distinct and potentially superior cardioprotective option.

**Table 2:** Clinical studies on β-blockers for cardiotoxicity prevention.

Study ID	Design	Cancer type	Intervention & duration	Chemotherapy	Outcomes
Kalay et al. 2006 (29)	RCT	Breast, Lymphoma, Other	Carvedilol 12.5 mg QD; 6 months	Adriamycin or Epirubicin	EF preserved (69.7%) vs. decline to 52.3% in control; *p* < 0.001
Kaya et al. 2012 (47)	Double-blind	Breast	Nebivolol 5 mg QD; 6 months	Doxorubicin	EF higher in treatment (63.8%) vs. placebo (57.5%); BNP ↑ only in placebo; *p* = 0.01
Noori et al. 2000 (28)	RCT	Breast	Propranolol, Metoprolol, Carvedilol; 8.5 months	Adriamycin	EF ↑ from 28% to 41% (BB) vs. 26% to 32% (control); *p* = 0.015
Rode et al. 2025 (15)	Cohort	Not reported	Max BB doses; 12 months	Not reported	↓ mortality and MACE by 57% (HR 0.43)
Seicean et al. 2014 (48)	RCT	Breast	Multiple BBs; 38.4 months	Doxorubicin	↓ HF incidence (HR 0.2; 95% CI, 0.1–0.5; *p* = 0.003)

Among the 55 total studies reviewed, 30 studies specifically examined the effects of *β*-blockers (Table 3), ACE inhibitors (Table 4), or combined treatment strategies (Table 5) on cardiac outcomes, demonstrating statistically significant benefits. Enalapril significantly reduced heart failure incidence and hospitalizations for patients with Hodgkin's and Non-Hodgkin's Lymphoma receiving DOX (434 vs. 518 patients, 20% risk reduction, *P* < 0.001) (26). A separate trial demonstrated a 16% reduction in all-cause mortality with enalapril (35.2% vs. 39.7%, *P* = 0.0036), with the most significant effect on progressive heart failure deaths (22% reduction, *P* < 0.05) (27) Treatment with β-blockers showed improvement of LVEF from 28% to 41% (*P* = .041) in breast cancer patients receiving Adriamycin, compared to an increase from 26% to 32% (*P* = .015) in controls (28). Carvedilol use in anthracycline-treated patients preserved LVEF at 69.7%, whereas the control group experienced a significant decline to 52.3% (*P* < 0.001) (29). The statistical significance of heart failure hospitalizations across these interventions including enalapril, β-blockers, and carvedilol is summarized in Table 6 which emphasizes the reproducibility of benefit across different trials, with multiple agents showing significant reductions in both HF incidence and hospitalization rates, reinforcing their clinical utility in cancer patients receiving anthracyclines.

**Table 3:** Clinical trials on ACE inhibitors for cardiotoxicity prevention.

Study ID	Design	Intervention & duration	Chemotherapy	Outcomes
Cardinale et al. 2006 (31)	RCT	Enalapril 20 mg/day; 12 months	Idarubicin	↓ EF decline (0% vs. 43% in control); *p* < 0.001
Okumura et al. 2002 (49)	RCT	Lisinopril 20 mg/kg/day; 1 month	Adriamycin	↓ mortality (44%–12%); improved cardiac function
Sacco et al. 2001 (50)	RCT	Zofenopril 15 mg/kg/day; 1.25 months	Doxorubicin	Prevented ECG changes; no impact on antitumor efficacy
SOLVD Investigators, 1991	Double-blind	Enalapril 2.5–20 mg/day; 41.4 months	Not reported	↓ mortality by 16% vs. placebo; ↓ HF hospitalization
SOLVD Investigators et al. 1992 (14)	Double-blind	Enalapril; 37.4 months	Not reported	↓ HF/death by 29%; ↓ HF hospitalization
Tokudome et al. 2000 (51)	RCT	Enalapril 5 mg BID; 1 month	Doxorubicin	↑ survival (36%–100%); ↓ LVEDP and stroke work index; *p* < 0.05

**Table 4:** Clinical studies involving multiple pharmacologic interventions.

Study ID	Design	Cancer type	Intervention & duration	Chemotherapy	Outcomes
Bosch et al. 2013 (7)	RCT	Leukemia	Enalapril + Carvedilol	Not reported	EF decline only in control group (*Δ* = −3.4%; *p* = 0.09); preserved EF in treatment group
Georgakopoulos et al. 2010 (26)	RCT	Lymphoma	Metoprolol + Enalapril; 36 months	Doxorubicin	↓ HF and hospitalization (*p* < 0.001); ↓ mortality by 20%, ↓ composite HF/death by 29%
Guglin et al. 2019 (12)	Double-blind, placebo-controlled	Breast	Lisinopril or Carvedilol; 24 months	Not reported	↓ event rate: placebo 47%, lisinopril 37%, carvedilol 31% (*p* = 0.009, 0.015)
Gulati et al. 2016 (6)	Double-blind, placebo-controlled	Breast	Candesartan + Metoprolol	Not reported	No interaction effect (*p* = 0.530); no LVEF decline with metoprolol
Heck et al. 2021 (35)	Double-blind, 2 × 2 factorial	Breast	Candesartan + Metoprolol; 23 months	Epirubicin	↓ LV end-diastolic volume (*p* = 0.021); ↓ strain (*p* = 0.046)
Livi et al. 2021 (13)	Phase 3 RCT	Breast	Ramipril + Bisoprolol; 12 months	Not reported	↓ LVEF loss vs. placebo (*p* = 0.01); ↑ GLS in all active arms(*p* < 0.001)
Pituskin et al. 2017 (32)	Double-blind, placebo-controlled	Breast	Perindopril + Bisoprolol; 6 months	Docetaxel + Carboplatin + Trastuzumab	↓ EF decline with trastuzumab; *p* = 0.001
Silber et al. 2020 (33)	Systematic Review & Meta-analysis	Breast	Various combinations; median 6 months	Doxorubicin + Trastuzumab	Pooled EF benefit (MD 3.57; 95% CI 1.04–6.09)
Wihandono et al. 2021 (52)	RCT	Breast	Lisinopril + Bisoprolol; 6 months	Trastuzumab	↓ EF decline in treatment group; *p* = 0.017

**Table 5:** Comparative efficacy of interventions on cardiac outcomes.

Intervention	Ejection Fraction (EF%)	HF Incidence	Survival
ACE inhibitors	EF Preserved (0% vs. 43% in controls) (8)	↓ HF incidence: 28%–16% (*p* < 0.005) (17)	↑ survival; 36%–100% (*p* = 0.02) (53)
*β*-blockers	EF Preserved (69.7% vs. 52.3% in control) (26)	↓ HF incidence: 28%–9% (*p* < 0.002) (17)	↑ survival; 63%–84% (*p* = 0.009) (19)
SGLT2 inhibitors	EF Preserved (64.0% vs. 59.8% in control) (54)	↓ HF incidence: 30%–20% (*p* = 0.025) (18)	↑ survival; 57%–91%; (*p* < 0.001) (18)

**Table 6:** Statistical significance of heart failure reduction by intervention.

Intervention	Outcome	Statistical Measure
Enalapril	↓ HF incidence and hospitalizations vs control (17)	Incidence: *p* < 0.001 Hospitalization: *p* < 0.001
Enalapril	↓ HF incidence and hospitalizations vs control (55)	Incidence: *p* = 0.0036 Hospitalization: *p* = 0.05
β-Blockers	↓ HF incidence and hospitalizations vs control (40)	Incidence: *p* = 0.041 Hospitalization: *p* = 0.041
Carvedilol	↓ HF incidence and hospitalizations vs control (26)	Incidence: *p* < 0.001 Hospitalization: *p* < 0.001

Echocardiographic data confirmed that the LVEF of breast cancer patients receiving DOX remained stable with carvedilol but significantly declined in controls (*P* < 0.001) (30). Similarly, patients with various types of cancer receiving Idarubicin who were treated with enalapril had a lower rate of LVEF decline, with 0% meeting the primary endpoint of >10% LVEF reduction, compared to 43% in the control group (*P* < 0.001) (31). Additionally, in breast cancer patients, bisoprolol attenuated anthracycline-induced LVEF decline (−1 ± 5%) compared to perindopril (−3% ± 4%) and placebo (−5% ± 5%) (*P* = .001) (32). These results, along with pooled data from meta-analyses, are summarized in Table 7, which outlines the effectiveness of various interventions in preventing LVEF decline, illustrate that certain interventions, including carvedilol, enalapril, and bisoprolol, offer statistically significant preservation of LVEF compared to control or placebo, particularly when initiated early in the treatment course.

**Table 7:** Effectiveness of cardioprotective interventions in preventing EF decline.

Intervention	Outcome	Statistical measure
Carvedilol	EF remained stable in Carvedilol group; declined in controls (37)	*p* < 0.001
Enalapril	Prevented >10% EF decline; high rate of decline in control group (8)	*p* < 0.001
Bisoprolol vs Perindopril vs Placebo	Bisoprolol more effective at attenuating EF decline (43)	*p* < 0.001
RAAS blockers, β-blockers, Aldosterone Antagonists	Prevented EF reduction across 11 studies (14)	MD 3.57; 95% CI 1.04–6.09

A meta-analysis confirmed that RAAS blockers, β-blockers, and aldosterone antagonists significantly prevented LVEF reduction in cancer patients receiving anthracyclines (MD 3.57, 95% CI 1.04–6.09) across 11 studies (33).

Several studies found no significant differences in LVEF or FS following anthracycline therapy, with cardiac function remaining within normal limits across treatment groups (34). A small decline in LVEF was observed over extended follow-up, but no significant between-group differences were detected: candesartan [−1.7% (95% CI, 0.5–2.8)] vs. no candesartan [−1.8% (95% CI, 0.6–3.0)] and metoprolol [−1.6% (95% CI, 0.4–2.7)] vs. no metoprolol [−1.9% (95% CI, 0.7–3.0)] (35). No significant interaction was observed between candesartan and metoprolol (*P* = 0.530), and metoprolol did not affect overall LVEF decline (6). Rates of early cardiotoxicity (metoprolol: 10%, enalapril: 12%, control: 15%, *P* = 0.12) and late cardiotoxicity (5%, 6%, and 8%, respectively, *P* = 0.18) did not reach statistical significance (36). In lymphoma patients receiving doxorubicin, no significant changes in echocardiographic variables were observed over 10 years, and none developed clinical heart failure. These non-significant or null findings are summarized in Table 8, which highlights interventions that did not produce statistically meaningful differences in cardiac outcomes.This table is essential for contextualizing the limitations of existing strategies, showing that not all agents confer benefit and underscoring the importance of patient selection and study design in future cardioprotection trials.

**Table 8:** Interventions without statistically significant differences in cardiac outcomes.

Intervention	Outcome	Statistical measure
Multiple treatment groups	No significant differences in LVEF or FS; cardiac function remained normal (10)	Not significant
Candesartan vs No candesartan; Metoprolol vs No metoprolol	Small LVEF decline over time; no between-group differences (21)	CIs overlapped for all comparisons
Candesartan and metoprolol interaction	No significant interaction effect (20)	*p* = 0.530
Metoprolol, enalapril, control	No statistical difference in early or late cardiotoxicity (36)	*p* = 0.12 (early), *p* = 0.18 (late)
Doxorubicin-treated lymphoma patients	No significant changes in echo variables over 10 years; no clinical HF (17)	Not significant

Notably, there were no studies found that included direct head-to-head comparisons between ACE inhibitors and β-blockers to SGLT2 Inhibitors.

## Discussion

Anthracycline-induced cardiotoxicity poses a significant obstacle in oncological management as it may compromise the long-term cardiovascular health of cancer survivors. Despite decades of research, identifying an effective prophylactic strategy remains yet to be fully elucidated. This systematic review of preclinical and clinical studies underscores three pharmacologic classes, SGLT2 inhibitors, ACE inhibitors, and β-blockers, as frontrunners in cardioprotection. However, the consistency of evidence, the mechanistic rationale, and translational potential vary across these agents.

SGLT2 inhibitors have consistently demonstrated cardioprotective efficacy across both preclinical and clinical studies. Rodent models of doxorubicin-induced cardiomyopathy show preserved or improved LVEF, attenuated myocardial fibrosis, and reduced oxidative injury. Large clinical trials such as CANVAS and DAPA-HF, suggest meaningful reductions in cardiovascular events and heart failure hospitalizations, even in non-diabetic populations. In comparison, ACE inhibitors and β-blockers have shown modest to moderate benefits in preserving cardiac function during anthracycline therapy. Enalapril and carvedilol, in particular, have been associated with reductions in LVEF decline ranging from 5% to 10%, as well as mitigation of structural cardiac changes on imaging. However, randomized trial data for these agents show variability in endpoints, some studies report significant improvements in LVEF and NT-proBNP levels, while others show non-significant between-group differences or benefits limited to surrogate markers rather than clinical events. The inconsistency in statistical significance and effect size across trials may reflect differences in dosing, timing, and patient selection.

The preclinical efficacy of SGLT2 inhibitors, marked by preserved LVEF and reduction in oxidative and inflammatory damage, appears more robust than the variable effects seen with ACE inhibitors and β-blockers. SGLT2 inhibitors may address mechanistic gaps induced by anthracycline's pharmacological effects, particularly in inflammation and myocardial energetics, that are not fully targeted by current treatment methods. While ACE inhibitors and β-blockers primarily act through neurohormonal pathways, reducing afterload and sympathetic activation, SGLT2 inhibitors exert broader effects, including attenuation of mitochondrial dysfunction, suppression of pro-inflammatory cytokines, and improvement of myocardial metabolism and energetics. These mechanisms may better counteract the multifactorial cardiac injury induced by anthracyclines, which involves oxidative stress, mitochondrial damage, and metabolic derangements.

However, despite their promise, important gaps remain, particularly a lack of long-term clinical data in patients receiving anthracycline chemotherapy. The optimal timing, duration, and dosing of SGLT2 inhibitors in this setting have yet to be clearly established. Furthermore, evidence is limited regarding their efficacy in specific high-risk subgroups, such as those with pre-existing cardiovascular disease or severely reduced baseline LVEF. Notably, a population-based cohort study of older adults with diabetes undergoing anthracycline therapy reported zero heart failure hospitalizations among those treated with SGLT2 inhibitors, compared to a rate of 2.1 per 100 person-years in the untreated group, though incident heart failure diagnoses did not reach statistical significance [Abdel-Qadir et al. (37)]. Future trials focused on these variables are essential to define the role of SGLT2 inhibitors in this context.

The findings summarized in Table 9 highlight the significant cardioprotective benefits of ACE inhibitors, β-blockers, and SGLT2 inhibitors in patients receiving anthracycline chemotherapy. Enalapril and carvedilol effectively prevented declines in ejection fraction, with enalapril showing complete prevention of significant LVEF reduction, while dapagliflozin demonstrated promising cardiac preservation in preclinical models. All three agents also reduced heart failure incidence, with dapagliflozin notably lowering HF rates and overall mortality compared to controls. The survival benefits observed—ranging from improved cardiotoxicity-free survival with carvedilol and lisinopril to mortality reductions with enalapril and dapagliflozin—support the clinical value of incorporating these therapies for cardioprotection. These results reinforce the potential role of these drug classes in mitigating anthracycline-induced cardiotoxicity and improving long-term cardiac outcomes in cancer patients.

**Table 9:** Preclinical and clinical outcomes of SGLT2 inhibitors in cardiotoxicity prevention.

Intervention	Outcome	Statistical Measure
Canagliflozin	↓ major CV events and kidney failure (31)	HR 0.68 (95% CI, 0.49–0.94); HR 0.85 (95% CI, 0.69–1.06)
SGLT2 inhibitors vs. Other drugs	↓ HF incidence vs non-SGLT2i treatments (28)	HR 0.61 (95% CI, 0.51–0.73); *p* < 0.001
Empagliflozin in doxorubicin-exposed mice	Preserved LV function vs EF decline in Doxorubicin-only (7)	*p* = 0.011
Empagliflozin + Doxorubicin vs. Doxorubicin-Only	↑ EF; ↓ HF incidence (44)	*p* < 0.001; *p* < 0.05
Empagliflozin + Doxorubicin vs. Doxorubicin-Only	EF and FS recovery observed (41)	*p* = 0.06
Empagliflozin + Doxorubicin vs. Doxorubicin-Only	Improved EF in treated group (46)	*p* = 0.007

Studies examining the potential utilization of SGLT2 inhibitors in the aforementioned clinical circumstance could be attributed to their distinct mechanistic profile, which includes reduced oxidative stress, anti-inflammatory activity, natriuresis, and improved myocardial energetics. These actions may address the pathophysiological consequences of anthracycline-induced cardiotoxicity not adequately targeted by ACE inhibitors and β-blockers. While the ACE inhibitors and β-blockers primarily act via RAAS and sympathetic inhibition (2), SGLT2 inhibitors may offer a more comprehensive cardioprotective effect.

Given their prophylactic effect, SGLT2 inhibitors may be utilized to prevent cardiomyopathy in high-risk populations—such as patients with reduced baseline LVEF or elevated NT-proBNP levels—undergoing anthracycline therapy. Early initiation could allow for detection of subclinical dysfunction allowing for timely intervention leading to improved cardiac outcomes and minimized long-term cardiac sequelae for patients undergoing anthracycline therapy.

### Clinical translation considerations

While SGLT2 inhibitors demonstrate robust cardioprotective effects in preclinical and cardiovascular settings, several considerations must be addressed before broad implementation in oncology care. First, the long-term safety of these agents in patients receiving chemotherapy remains incompletely understood, particularly given the risks of immunosuppression, renal impairment, or volume depletion. Second, the potential impact of SGLT2 inhibitors on tumor biology or treatment response has not been systematically evaluated. It remains unclear whether these agents could interfere with chemotherapy efficacy, alter tumor metabolism, or influence recurrence risk.

Third, potential drug–drug interactions with common chemotherapeutics, targeted agents, or supportive medications must be carefully examined. For example, co-administration with nephrotoxic agents or diuretics may heighten the risk of renal or electrolyte disturbances. Finally, adherence poses a critical challenge in oncology populations, where polypharmacy, fatigue, and treatment-related side effects can undermine consistent use. Future clinical trials should therefore incorporate not only efficacy and safety endpoints but also real-world feasibility measures such as patient adherence, quality of life, and overall treatment burden.

### Recommendations

Future research should prioritize well-designed prospective trials that directly compare these therapeutic agents. Key proposed study designs include:

(1) A randomized controlled trial comparing ACE inhibitors and β-blockers against SGLT2 inhibitors and placebo in cancer patients without pre-existing heart failure who are receiving anthracycline therapy, with endpoints including LVEF decline, GLS change, and NT-proBNP levels.
(2) A combination therapy trial assessing whether dual (ACE inhibitors + β-blockers) or triple (ACE inhibitors + β-blockers + SGLT2 inhibitors) regimens offer synergistic benefit compared to SGLT2 inhibitor monotherapy.
(3) A risk-adapted strategy trial, enrolling patients based on NT-proBNP, LVEF, or GLS thresholds, to determine the efficacy of early prophylactic intervention with SGLT2 inhibitors in high-risk subgroups undergoing anthracycline therapy.

### Limitations of included studies

Despite the breadth of evidence reviewed, several limitations should be noted. Many clinical trials and observational studies were underpowered due to small sample sizes, making it difficult to detect long-term or rare outcomes such as late-onset heart failure. Short follow-up durations may further underestimate the true incidence of delayed cardiotoxicity, which is a known risk with anthracycline therapy.

Substantial heterogeneity in study design, including differences in patient populations, chemotherapy protocols, and outcome measures, makes direct comparisons challenging. Inconsistent definitions of cardiotoxicity, variable use of cardiac biomarkers, and limited application of modern imaging techniques such as cardiac MRI or GLS reduce the generalizability of results.

Additionally, much of the mechanistic data for SGLT2 inhibitors comes from preclinical rodent models that do not fully capture the complexity of human oncology patients, who often have comorbidities like diabetes or pre-existing cardiovascular disease. Few studies stratified outcomes by important clinical factors such as baseline cardiovascular risk, diabetes status, or sex, further limiting the applicability of findings to diverse patient subgroups.

Finally, the absence of head-to-head comparisons between SGLT2 inhibitors and established agents such as ACE inhibitors and β-blockers underscores the need for larger, rigorously designed trials with standardized endpoints and longer follow-up to clarify the role of these emerging cardioprotective strategies.

## Conclusion

This review evaluates the potential cardioprotective role of SGLT2 inhibitors in mitigating anthracycline-induced cardiotoxicity. ACE inhibitors and β-blockers, well-established in heart failure management, continue to show relevance in this setting, especially for patients with preexisting cardiovascular risk. However, the lack of head-to-head comparative trials limits the ability to determine an optimal clinical strategy. Future research must prioritize randomized studies that evaluate these agents in parallel and in combination, using standardized anthracycline regimens and unified cardiac endpoints. As cancer survival improves, preventing long-term cardiac complications becomes increasingly vital. Closing these evidence gaps will be essential to guiding therapy, shaping cardio-oncology guidelines, and safeguarding the cardiovascular health of cancer survivors.

## Data Availability

The original contributions presented in the study are included in the article/Supplementary Material, further inquiries can be directed to the corresponding author.

## References

[1] Swain SM Whaley FS Ewer MS. Congestive heart failure in patients treated with doxorubicin: a retrospective analysis of three trials. Cancer. (2003) 97(11):2869–79. 10.1002/cncr.11407 12767102

[2] Zhang S Liu X Bawa-Khalfe T Lu L-S Lyu YL Liu LF Identification of the molecular basis of doxorubicin-induced cardiotoxicity. Nat Med. (2012) 18:1639–42. 10.1038/nm.2919 23104132

[3] Minotti G Menna P Salvatorelli E Cairo G Gianni L. Anthracyclines: molecular advances and pharmacologic developments in antitumor activity and cardiotoxicity. Pharmacol Rev. (2004) 56(2):185–229. 10.1124/pr.56.2.6 15169927

[4] Wiseman LR Spencer CM. Dexrazoxane: a review of its use as a cardioprotective agent in patients receiving anthracycline-based chemotherapy. Drugs. (2012) 56(3):385–403. 10.2165/00003495-199856030-00009 9777314

[5] Swedberg K Cleland JG Dargie H Drexler H Follath F Komajda M Guidelines for the diagnosis and treatment of chronic heart failure: executive summary (update 2005): the task force for the diagnosis and treatment of chronic heart failure of the European Society of Cardiology. Eur Heart J. (2005) 26:1115–40. 10.1093/eurheartj/ehi204 15901669

[6] Gulati G Heck SL Ree AH Hoffmann P Schulz-Menger J Fagerland MW Prevention of cardiac dysfunction during adjuvant breast cancer therapy (PRADA): a 2×2 factorial, randomized, placebo-controlled, double-blind clinical trial of candesartan and metoprolol. Eur Heart J. (2016) 37:1671–80. 10.1093/eurheartj/ehw022 26903532 PMC4887703

[7] Bosch X Rovira M Sitges M Domènech A Ortiz-Pérez JT de Caralt TM Enalapril and carvedilol for preventing chemotherapy-induced left ventricular dysfunction in patients with malignant haemopathies: the OVERCOME trial. Eur Heart J. (2013) 34(30):2355–62. 10.1016/j.jacc.2013.02.072 23583763

[8] Fitchett D Zinman B Wanner C Lachin J Hantel S Salsali A Heart failure outcomes with empagliflozin in patients with type 2 diabetes at high cardiovascular risk: results of the EMPA-REG OUTCOME® trial. Eur Heart J. (2016) 37(19):1529–40. 10.1093/eurheartj/ehv728 PMC4872285 26819227

[9] Yang CC Chen YT Wallace CG Chen KH Cheng BC Sung PH Early administration of EMPA preserved heart function in cardiorenal syndrome in rat. Biomed Pharmacother. (2019) 109:658–70. 10.1016/j.biopha.2018.10.095 30404073

[10] Lopaschuk GD Verma S. Mechanisms of cardiovascular benefits of sodium glucose co-transporter 2 (SGLT2) inhibitors: a state-of-the-art review. JACC Basic Transl Sci. (2020) 5(6):632–44. 10.1016/j.jacbts.2020.02.004 32613148 PMC7315190

[11] Sterne JAC Hernán MA Reeves BC Savović J Berkman ND Viswanathan M ROBINS-I: a tool for assessing risk of bias in non-randomised studies of interventions. Br Med J. (2016) 355:i4919. 10.1136/bmj.i4919 27733354 PMC5062054

[12] Guglin M Krischer J Tamura R Fink A Bello-Matricaria L McCaskill-Stevens W Randomized trial of lisinopril versus carvedilol to prevent trastuzumab cardiotoxicity in patients with breast cancer. J Am Coll Cardiol. (2019) 73(23):2859–68. 10.1016/j.jacc.2019.03.495 31171092 PMC6557296

[13] Livi L Barletta G Martella F Saieva C Desideri I Bacci C Cardioprotective strategy for patients with nonmetastatic breast cancer who are receiving an anthracycline-based chemotherapy: a randomized clinical trial. JAMA Oncol. (2021) 7(10):1544–9. 10.1001/jamaoncol.2021.3395 34436523 PMC8391772

[14] SOLVD Investigators, Yusuf S Pitt B Davis CE Hood WB Cohn JN. Effect of enalapril on mortality and the development of heart failure in asymptomatic patients with reduced left ventricular ejection fractions. N Engl J Med. (1992) 327(10):685–91. 10.1056/NEJM199209033271003 1463530

[15] Rode F Pavlović N Jordan A Radić M Lisičić A Sokol Tomić S The use of *β*-blockers for heart failure with reduced ejection fraction in the era of SGLT2 inhibitors—are we still afraid to up-titrate? Heart Vessels. (2025) 40:797–804. 10.1007/s00380-025-02525-7 39934336

[16] Page MJ McKenzie JE Bossuyt PM Boutron I Hoffmann TC Mulrow CD The PRISMA 2020 statement: an updated guideline for reporting systematic reviews. BMJ. (2021) 372:n71. 10.1136/bmj.n71 33782057 PMC8005924

[17] Mahaffey KW Jardine MJ Bompoint S Cannon CP Neal B Heerspink HJL Canagliflozin and cardiovascular and renal outcomes in type 2 diabetes mellitus and chronic kidney disease in primary and secondary cardiovascular prevention groups. Circulation. (2019) 140(9):739–50. 10.1161/CIRCULATIONAHA.119.042007 31291786 PMC6727954

[18] Kosiborod M Cavender MA Fu AZ Wilding JP Khunti K Holl RW Lower risk of heart failure and death in patients initiated on sodium-glucose cotransporter-2 inhibitors versus other glucose-lowering drugs: the CVD-REAL study. Circulation. (2017) 136(3):249–59. 10.1161/CIRCULATIONAHA.117.029190 28522450 PMC5515629

[19] Byrne NJ Parajuli N Levasseur JL. Empagliflozin prevents worsening of cardiac function in an experimental model of pressure overload-induced heart failure. J Am Coll Cardiol Basic Trans Science. (2017) 1:347–54. 10.1016/j.jacbts.2017.07.003 PMC6034464 30062155

[20] Quagliariello V De Laurentiis M Rea D Barbieri A Monti MG Carbone A The SGLT-2 inhibitor empagliflozin improves myocardial strain, reduces cardiac fibrosis and pro-inflammatory cytokines in non-diabetic mice treated with doxorubicin. Cardiovasc Diabetol. (2021) 20:150. 10.1186/s12933-021-01324-2 34301253 PMC8305868

[21] Oh CM Cho S Jang JY Kim H Chun S Choi M Cardioprotective potential of an SGLT2 inhibitor against doxorubicin-induced heart failure. Korean Circ J. (2019) 49(12):1183–95. 10.4070/kcj.2019.0180 31456369 PMC6875592

[22] Sabatino J De Rosa S Tammè L Iaconetti C Sorrentino S Polimeni A EMPA prevents doxorubicin-induced myocardial dysfunction. Cardiovasc Diabetol. (2020) 19(1):66. 10.1186/s12933-020-01040-5 32414364 PMC7229599

[23] Ulusan S Gülle K Peynirci A Sevimli M Karaibrahimoğlu A. Dapagliflozin may protect against doxorubicin-induced cardiotoxicity. Anatol J Cardiol. (2023) 27(6):339–47. 10.14744/AnatolJCardiol.2023.2825 37257007 PMC10250773

[24] Fath AR Aglan M Aglan A Chilton RJ Trakhtenbroit A Al-Shammary OA Cardioprotective potential of sodium-glucose cotransporter-2 inhibitors in patients with cancer treated with anthracyclines: an observational study. Am J Cardiol. (2024) 222:175–82. 10.1016/j.amjcard.2024.04.032 38692401

[25] Bhatti AW Patel R Dani SS Khadke S Makwana B Lessey C SGLT2i and primary prevention of cancer therapy–related cardiac dysfunction in patients with diabetes. JACC: CardioOncology. (2024) 6(6):863–75. 10.1016/j.jaccao.2024.05.006 39801650 PMC11711834

[26] Georgakopoulos P Roussou P Matsakas E Karavidas A Anagnostopoulos N Marinakis T Cardioprotective effect of metoprolol and enalapril in doxorubicin-treated lymphoma patients: a prospective, parallel-group, randomized, controlled study with 36-month follow-up. Am J Hematol. (2010) 85(12):894–6. 10.1002/ajh.21840 20872550

[27] SOLVD Investigators. Effect of enalapril on survival in patients with reduced left ventricular ejection fractions and congestive heart failure. N Engl J Med. (1991) 325(5):293–302. 10.1056/NEJM199108013250501 2057034

[28] Noori A Lindenfeld J Wolfel E Ferguson D Bristow MR Lowes BD. Beta-blockade in adriamycin-induced cardiomyopathy. J Card Fail. (2000) 6:115–9. 10908085

[29] Kalay N Basar E Ozdogru I Er O Cetinkaya Y Dogan A Protective effects of carvedilol against anthracycline-induced cardiomyopathy. J Am Coll Cardiol. (2006) 48(11):2258–62. 10.1016/j.jacc.2006.06.068 17161256

[30] Nabati M Janbabai G Baghyari S Esmaili K Yazdani J. Cardioprotective effects of carvedilol in inhibiting doxorubicin-induced cardiotoxicity. J Cardiovasc Pharmacol. (2017) 69(5):279–85. 10.1097/FJC.0000000000000470 28141699

[31] Cardinale D Colombo A Sandri MT Lamantia G Colombo N Civelli M Prevention of high-dose chemotherapy-induced cardiotoxicity in high-risk patients by angiotensin-converting enzyme inhibition. Circulation. (2006) 114(24):2474–81. 10.1161/CIRCULATIONAHA.106.633050 17101852

[32] Pituskin E Mackey JR Koshman S Jassal D Pitz M Haykowsky MJ Multidisciplinary approach to novel therapies in cardio-oncology research (MANTICORE 101-breast): a randomized trial for the prevention of anthracycline-induced cardiotoxicity by carvedilol. J Clin Oncol. (2017) 35(7):820–8. 10.1200/JCO.2016.68.7830 27893331

[33] Gao Y Wang R Jiang J Hu J Li H Wang Y. ACEI/ARB and beta-blocker therapies for preventing cardiotoxicity of antineoplastic agents in breast cancer: a systematic review and meta-analysis. Heart Fail Rev. (2023) 28(6):1405–15. 10.1007/s10741-023-10328-z 37414918 PMC10575808

[34] Elitok A Oz F Cizgici AY Kilic L Ciftci R Sen F Effect of carvedilol on silent anthracycline-induced cardiotoxicity assessed by strain imaging: a prospective randomized controlled study with six-month follow-up. Cardiol J. (2014) 21(5):509–15. 10.5603/CJ.a2013.0150 24142687

[35] Heck SL Mecinaj A Ree AH Hoffmann P Schulz-Menger J Fagerland MW Prevention of cardiac dysfunction during adjuvant breast cancer therapy (PRADA): extended follow-up of a 2×2 factorial, randomized, placebo-controlled, double-blind clinical trial of candesartan and metoprolol. Circulation. (2021) 143(25):2431–40. 10.1161/CIRCULATIONAHA.121.054698 33993702 PMC8212877

[36] Zubair M Khan A Nadeem M Ali S. A randomized controlled trial of metoprolol versus enalapril for the prevention of chemotherapy-induced cardiotoxicity in lymphoma patients. J Clin Oncol. (2022) 40(6):514–22. 10.1200/JCO.22.00678

[37] Abdel-Qadir H Carrasco R Austin PC Chen Y Zhou L Fang J The association of sodium-glucose cotransporter 2 inhibitors with cardiovascular outcomes in anthracycline-treated patients with cancer. JACC CardioOncol. (2023) 5(3):318–28. 10.1016/j.jaccao.2023.04.006 37397088 PMC10308059

[38] OpenAI. ChatGPT. (May 2025 version) [Large language model]. San Francisco, CA: OpenAI (2025). Available online at: https://chat.openai.com/

[39] Anker SD Butler J Filippatos G Ferreira JP Bocchi E Böhm M Empagliflozin in heart failure with a preserved ejection fraction. N Engl J Med. (2021) 385(16):1451–61. 10.1056/NEJMoa2107038 34449189

[40] Das BB Niu J. A systematic review and meta-analysis of the safety and efficacy of SGLT2 inhibitors in chronic heart failure in ACHD patients. Am J Cardiovasc Drugs. (2025) 25(2):231–40. 10.1007/s40256-024-00697-7 39621203 PMC11811263

[41] Mahaffey KW Neal B Perkovic V de Zeeuw D Fulcher G Erondu N Canagliflozin for primary and secondary prevention of cardiovascular events: results from the CANVAS program (Canagliflozin Cardiovascular Assessment Study). Circulation. (2018) 137:323–34. 10.1161/CIRCULATIONAHA.117.032038 29133604 PMC5777572

[42] McMurray JJV Solomon SD Inzucchi SE Køber L Kosiborod MN Martinez FA Dapagliflozin in patients with heart failure and reduced ejection fraction. N Engl J Med. (2019) 381(21):1995–2008. 10.1056/NEJMoa1911303 31535829

[43] Shi X Verma S Yun J Brand-Arzamendi K Singh KK Liu X Effect of empagliflozin on cardiac biomarkers in a zebrafish model of heart failure: clues to the EMPA-REG OUTCOME trial? Mol Cell Biochem. (2017) 433:97–102. 10.1007/s11010-017-3018-2 28391552

[44] Zannad F Ferreira JP Pocock SJ Anker SD Butler J Filippatos G SGLT2 inhibitors in patients with heart failure with reduced ejection fraction: a meta-analysis of the EMPEROR-reduced and DAPA-HF trials. Lancet. (2020) 396(10254):819–29. 10.1016/S0140-6736(20)31824-9 32877652

[45] Zelniker TA Wiviott SD Raz I Im K Goodrich EL Bonaca MP SGLT2 inhibitors for primary and secondary prevention of cardiovascular and renal outcomes in type 2 diabetes: a systematic review and meta-analysis of cardiovascular outcome trials. Lancet. (2019) 393:31–3. 10.1016/S0140-6736(18)32590-X 30424892

[46] Zinman B Wanner C Lachin JM Fitchett D Bluhmki E Hantel S Empagliflozin, cardiovascular outcomes, and mortality in type 2 diabetes. N Engl J Med. (2015) 373(22):2117–28. 10.1056/NEJMoa1504720 26378978

[47] Kaya MG Ozkan M Gunebakmaz O Akkaya H Kaya EG Akpek M Protective effects of nebivolol against anthracycline-induced cardiomyopathy: a randomized control study. Int J Cardiol. (2012) 157(2):181–4. 10.1016/j.ijcard.2012.06.023 22727976

[48] Seicean S Seicean A Alan N Plana JC Budd GT Marwick TH Cardioprotective effect of beta-adrenoceptor blockade in patients with breast cancer undergoing chemotherapy: a cardiac MRI study. Eur Heart J Cardiovasc Imaging. (2014) 15(8):851–8. 10.1161/CIRCHEARTFAILURE.112.000055

[49] Okumura K Jin D Takai S Miyazaki M. Beneficial effects of angiotensin-converting enzyme inhibition in Adriamycin-induced cardiomyopathy in hamsters. Jpn J Pharmacol. (2002) 88(2):183–8. 10.1254/jjp.88.183 11928719

[50] Sacco G Bigioni M Evangelista S Goso C Manzini S Maggi CA. Cardioprotective effects of zofenopril, a new angiotensin-converting enzyme inhibitor, on doxorubicin-induced cardiotoxicity in the rat. Eur J Pharmacol. (2001) 414(1):71–8. 10.1016/S0014-2999(00)01070-X 11230997

[51] Tokudome T Mizushige K Noma T Manabe K Murakami K Tsuji T Prevention of doxorubicin (Adriamycin)-induced cardiomyopathy by simultaneous administration of angiotensin-converting enzyme inhibitor assessed by acoustic densitometry. J Cardiovasc Pharmacol. (2000) 36(3):361–8. 10.1097/00005344-200009000-00003 10975594

[52] Wihandono A Azhar Y Abdurahman M Hidayat S. The role of lisinopril and bisoprolol to prevent anthracycline-induced cardiotoxicity in locally advanced breast cancer patients. Asian Pac J Cancer Prev. (2021) 22(9):2847–53. 10.31557/APJCP.2021.22.9.2847 34582653 PMC8850900

[53] Kalam K Marwick TH. Role of cardioprotective therapy for prevention of cardiotoxicity with chemotherapy: a systematic review and meta-analysis. Eur J Cancer. (2013) 49(13):2900–9. 10.1016/j.ejca.2013.04.030 23706982

[54] de Nigris F Rienzo M Schiano C Fiorito C Casamassimi A Napoli C. Prominent cardioprotective effects of third generation beta blocker nebivolol against anthracycline-induced cardiotoxicity using the model of isolated perfused rat heart. Eur J Cancer. (2008) 44:334–40. 10.1016/j.ejca.2007.12.010 18194856

[55] Neal B Perkovic V Mahaffey KW de Zeeuw D Fulcher G Erondu N Canagliflozin and cardiovascular and renal events in type 2 diabetes. N Engl J Med. (2017) 377(7):644–57. 10.1056/NEJMoa1611925 28605608

[56] Wiviott SD Raz I Bonaca MP Mosenzon O Kato ET Cahn A Dapagliflozin and cardiovascular outcomes in type 2 diabetes (DECLARE–TIMI 58 trial). N Engl J Med. (2019) 380:347–57. 10.1056/NEJMoa1812389 30415602

